# The animal trypanosomiases and their chemotherapy: a review

**DOI:** 10.1017/S0031182016001268

**Published:** 2016-10-10

**Authors:** FEDERICA GIORDANI, LIAM J. MORRISON, TIM G. ROWAN, HARRY P. DE KONING, MICHAEL P. BARRETT

**Affiliations:** 1 Wellcome Trust Centre for Molecular Parasitology, Institute of Infection, Immunity and Inflammation, College of Medical, Veterinary and Life Sciences, University of Glasgow, Glasgow G12 8TA, UK; 2 Roslin Institute, Royal (Dick) School of Veterinary Studies, University of Edinburgh, Easter Bush, Midlothian EH25 9RG, UK; 3 Global Alliance for Livestock Veterinary Medicines (GALVmed), Doherty Building, Pentlands Science Park, Bush Loan, Edinburgh EH26 0PZ, UK

**Keywords:** animal trypanosomiases, veterinary trypanocide, drug resistance, *Trypanosoma congolense*, *Trypanosoma vivax*, *Trypanosoma brucei*

## Abstract

Pathogenic animal trypanosomes affecting livestock have represented a major constraint to agricultural development in Africa for centuries, and their negative economic impact is increasing in South America and Asia. Chemotherapy and chemoprophylaxis represent the main means of control. However, research into new trypanocides has remained inadequate for decades, leading to a situation where the few compounds available are losing efficacy due to the emergence of drug-resistant parasites. In this review, we provide a comprehensive overview of the current options available for the treatment and prophylaxis of the animal trypanosomiases, with a special focus on the problem of resistance. The key issues surrounding the main economically important animal trypanosome species and the diseases they cause are also presented. As new investment becomes available to develop improved tools to control the animal trypanosomiases, we stress that efforts should be directed towards a better understanding of the biology of the relevant parasite species and strains, to identify new drug targets and interrogate resistance mechanisms.

## INTRODUCTION

**Fig. 1.**
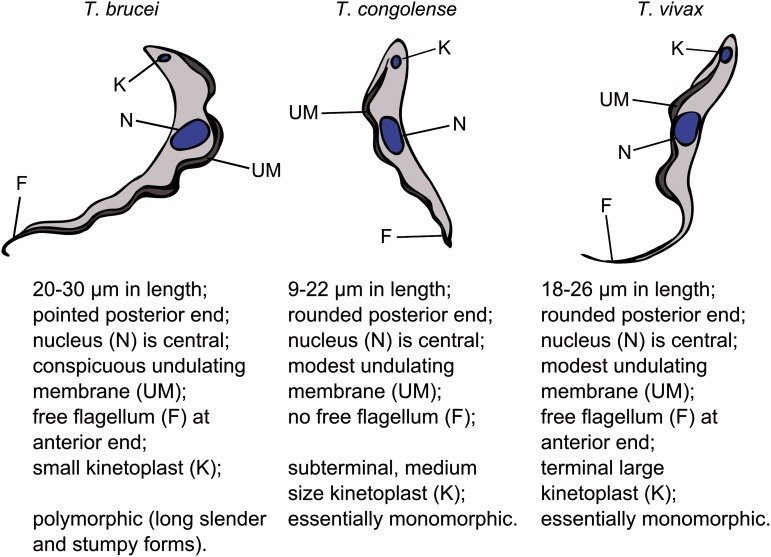
Morphological characteristics of the bloodstream form trypomastigote of the three most important livestock trypanosomes. *T. brucei* group trypanosomes (*T. b. brucei, T. b. evansi, T. b. equiperdum*) are morphologically indistinguishable (with the exception of the non-proliferative stumpy-form in *T. b. brucei*). The trypomastigote is the disease-relevant form and the target of therapy.

The animal trypanosomiases (or trypanosomoses) include a variety of wasting diseases caused by unicellular protozoan parasites of the genus *Trypanosoma* (order Kinetoplastida). All relevant animal pathogenic trypanosomes (*T. vivax* – subgenus *Duttonella, T. congolense* – subgenus *Nannomonas* and *T. brucei* spp. – subgenus *Trypanozoon*) (Fig. 1) belong to the Salivaria group (Haag *et al.* 1998), so-called because their transmission to the vertebrate host occurs principally via the infected saliva of blood-sucking insects. Most valuable domestic livestock (bovines, ovines, caprines, equids, camelids and suids) are susceptible to infection with one or more of these *Trypanosoma* species. This can lead to acute and/or chronic forms of wasting disease, causing high morbidity, mortality and infertility in the absence of treatment (Leach and Roberts, 1981; Connor, 1992). By affecting agricultural production and animal husbandry, the animal trypanosomiases have a high economic and social impact in vast areas of the tropics and subtropics where transmission occurs. Africa has historically suffered the greatest burden (Steverding, 2008), but the negative effects are also increasing in South America and South-East Asia, where unrestricted animal movements favour the spread of some trypanosome species.

Chemotherapy and chemoprophylaxis represent the mainstay of animal trypanosomiases control, ensuring animal health and production in enzootic countries. However, the available veterinary trypanocides (Table 1) are inadequate and outmoded. Only six compounds are currently licensed, and their narrow therapeutic indices restrict their use, especially when even low-level resistance arises. By far, the most usage is of two compounds, diminazene aceturate and isometamidium chloride, largely applied against animal trypanosomiases in Africa (Holmes *et al.* 2004), with suramin also being relatively widely used to treat *T. b. evansi* infections. Worryingly, an increasing number of reports of resistance to this handful of existing chemicals, particularly diminazene and isometamidium, indicate their future utility to be in jeopardy (Geerts *et al.* 2001; Delespaux and de Koning, 2007).

**Table 1.** Currently available veterinary trypanocides.

Name	Trade names^a^	Structure	Administration route	Action	Dosage (mg Kg^−1^)^b^	Indication/animal	Adverse effects/other information	Treatment of relapses
Diminazene aceturate	Berenil, Veriben, Pirocide, Ganaseg, Azidin, Trypan	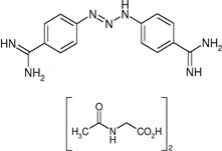	IM, SC	T	3·5 (up to 8 for resistant trypanosomes, 5–10 for *T. b. evansi*)	*T. congolense, T. vivax* (less active on *T. b. brucei, T. b. evansi*)/ Cattle, sheep, goats, dogs	Toxic to horses, donkeys, dogs and camels. Also babesicidal	Isometamidium chloride
Homidium bromide Homidium chloride	Ethidium Novidium	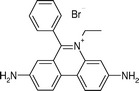	IM (deep, cattle), IV (sheep, goats, pigs)	T, (P)	1	*T. vivax, T. congolense* (less active on *T. b. brucei*)/ Cattle, sheep, goats, pigs	IM toxic to horses. Potentially carcinogenic	Diminazene aceturate, Isometamidium chloride
Isometamidium chloride	Trypamidium, Samorin, Veridium, Securidium	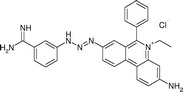	IM (deep)	P, T	0·25–1 (T), 0·5–1 (P)	*T. congolense, T. vivax* (less active on *T. b. brucei, T. b. evansi*)/ Cattle, sheep, goats, horses, camels	Toxic above 2 mg Kg^–1^. Avoid subcutaneous administration. Highly irritant. Possible local reactions in cattle.	Diminazene aceturate
Quinapyramine sulphate Quinapyramine sulphate:chloride (3:2 w/w)	Antrycide, Trypacide, Noroquin, Quintrycide, Tribexin, Triquin-S, M7555, Trypacide prosalt	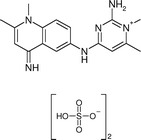	SC	T P	3–5 (T) (20–40 for *T. simiae*) (Camels, horses, pigs, dogs: dose divided and given at 6 h intervals), 7·4 (P)	*T. b. evansi, T. vivax, T. congolense, T. brucei, T. b. equiperdum, T. simiae*/ Camels, horses, pigs, dogs, cattle (discouraged)	Toxic at high doses. Fast resistance acquisition	Isometamidium chloride, Suramin sodium
Suramin sodium	Naganol, Bayer 205, Germanin	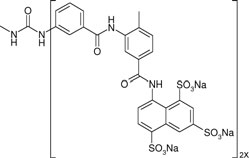	IV	T (P)	10 (horses: 3 doses/1 week)	*T. b. evansi, T. b. brucei, T. b. equiperdum*/ Camels, horses	IM can cause severe necrosis at injection site. May be toxic to horses	Quinapyramine sulphate
Melarsomine dihydrochloride	Cymelarsan, Mel Cy	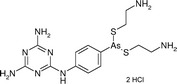	SC, IM	T	0·25–0·5 (0·5 for cattle)	*T. b. evansi, T. b. brucei, T. b. equiperdum*/ Camels, buffalo, goats, pigs, horses, cattle		

IM, intramuscular; IV, intravenous; SC, subcutaneous; T, therapeutic action; P, prophylactic action. *Note.* Products used in animals producing meat or milk for human consumption should only be used in full compliance with product labels including withdrawal periods.

a The list of the trade names is not complete.

b Dosages are for single administration except were stated otherwise.

It has been estimated that as many as 35 million doses of trypanocides are used annually in sub-Saharan Africa alone (Holmes, 2013), which represents a figure suitable to treat only around one-third of the cattle at risk (Swallow, 2000). Inclusion of trypanocides sold informally in the African market may substantially increase the total number of doses sold annually, which may be as high as 70 million doses (Frans van Gool, personal communication, 2015). Despite this demand, the high costs of drug development and the low anticipated profit from the sale of chemotherapeutics in developing countries have disincentivized commercial pharmaceutical investments in this field (Connor, 1992). In recent years, a public–private partnership, GALVmed (Global Alliance for Livestock Veterinary Medicines), supported by funding from the Bill & Melinda Gates Foundation and the UK Department for International Development, has emerged to fill the gap, and has committed to the development of new therapeutic and prophylactic trypanocidal drugs (http://www.galvmed.org/en/). However, even in the best case scenario, a novel licensed compound is unlikely to be available for several years yet; hence the rational, correct use of the trypanocides already available is of paramount importance.

## THE ANIMAL TRYPANOSOMIASES: DISTRIBUTION, TRANSMISSION, HOSTS, PATHOLOGY AND ECONOMIC IMPACT

### Animal African trypanosomiasis (AAT, nagana)

**Fig. 2.**
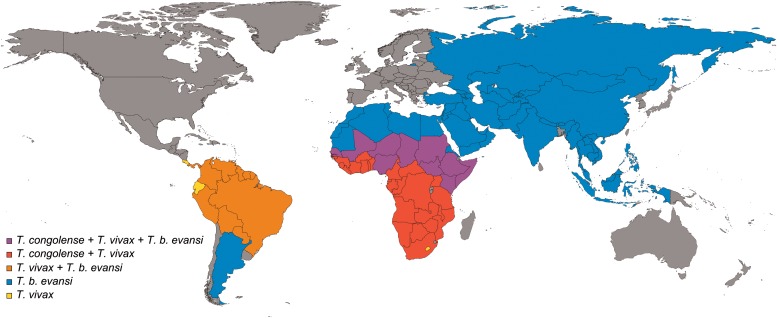
Countries where the most important livestock trypanosomes are present. Modified from (Auty *et al.* 2015), based on PubMed search and including countries where data were not available and parasite presence is inferred. To note that the real geographical distribution in some countries is limited (as, for example, for *T. congolense* in South Africa, Namibia and Botswana and for *T. b. evansi* in Russia). Cases of eradicated outbreaks of *T. b. evansi* in Europe (i.e. in France) are not indicated.

AAT [also called nagana, from the Zulu word ‘N'gana’ which means ‘powerless/useless’ (Steverding, 2008)], is caused by trypanosome species *T. congolense, T. vivax* and, to a lesser extent, *T. brucei* spp. (Fig. 1). The disease is widespread in sub-Saharan Africa (Fig. 2), where it is cyclically transmitted by the tsetse fly (*Glossina* spp.), the same vector responsible for the transmission of human-infective trypanosomes (*T. brucei gambiense* and *T. b. rhodesiense*, the aetiological agents of human African trypanosomiasis, HAT, or sleeping sickness) (Barrett *et al.* 2003). In animals, tsetse flies can also transmit trypanosomes mechanically when they begin a blood meal on an infected host and end it on another one, provided that the time between the two meals is short enough to ensure survival of parasites in the insect mouthparts, as shown in experimental infections in goats (Moloo *et al.* 2000). Unlike other trypanosomes, *T. vivax* does not multiply in the tsetse midgut, but remains confined to the insect proboscis, where it completes its short life cycle (Gardiner, 1989). This is the reason why this species can also be transmitted mechanically by other haematophagous flies, in particular horseflies (*Tabanus* spp.) and stable flies (*Stomoxys* spp.). Mechanical transmission has allowed *T. vivax* to spread far beyond the limits of the African tsetse belt: this parasite is now established in Mauritius and in 13 South American countries (Fig. 2), where it probably arrived in the 18th or 19th century via infected Zebu cattle exported from West Africa (Jones and Davila, 2001; Osorio *et al.* 2008), an origin corroborated by phylogenetic studies (Cortez *et al.* 2006). Although *T. vivax* remains enzootic in South America primarily due to mechanical transmission, other potential modes of transmission include perinatal and iatrogenic routes or via alternative, as yet unidentified vectors (Osorio *et al.* 2008). This lack of definitive knowledge greatly hampers the implementation of surveillance and control strategies (Jones and Davila, 2001). Non-tsetse transmitted *T. vivax* infection in cattle is also recognized in parts of Africa, for example in regions of Ethiopia, Chad and Sudan (Ahmed *et al.* 2016). Mechanical transmission of *T. congolense* has been shown under experimental conditions (Desquesnes and Dia, 2003) and can therefore not be excluded from contributing to its spread in Africa (Desquesnes *et al.* 2009).

The host range is wide (Uilenberg, 1998). *Trypanosoma congolense* is considered the most pathogenic trypanosome in cattle (followed by *T. vivax*), but it also causes infections in horses, sheep, goats, pigs and dogs. Apart from bovines, *T. vivax* can affect sheep, goats, horses and camels (Osorio *et al.* 2008). *Trypanosoma b. brucei* is found in various domestic ungulates but it is particularly virulent in dogs, camels and horses, the latter often succumbing to infection within a few months in the absence of treatment. In areas where more than one trypanosome species is present, mixed infections in domestic animals are often encountered (Kihurani *et al.* 1994; Auty *et al.* 2008; Biryomumaisho *et al.* 2013; Takeet *et al.* 2013; Moti *et al.* 2015) and modern molecular techniques (Desquesnes and Davila, 2002) facilitate speciation. Many wild animal species in Africa also host one or more trypanosome species and can serve as reservoirs for both human and domestic animal infective trypanosomes (Mulla and Rickman, 1988; Auty *et al.* 2012). Similarly, wild South American fauna can harbour *T. vivax* and act as reservoir of infection (Osorio *et al.* 2008).

Belonging to the same *Nannomonas* subgenus as *T. congolense, T. simiae* is the only trypanosome species to be extremely pathogenic to pigs, which represent the main host, although other domestic species can harbour the parasite (Joshua and Kayit, 1984; Salim *et al.* 2014). In pigs, *T. simiae* causes a hyperacute, often fatal infection, with death often occurring within 48 h of the appearance of symptoms (Leach and Roberts, 1981). For this reason, chemoprophylaxis is preferred to curative treatment.

The pathogenicity of trypanosomal infections varies considerably depending on several factors, including parasite-related aspects (species and virulence), host (species, breed, age, immunological status, nutritional status, presence of co-infection and physical condition), vector (species, density, infection rate and host preference), epidemiological situation (endemic or epidemic) and the environment (e.g. the availability of food and water and the season) (Leach and Roberts, 1981; Van den Bossche and Delespaux, 2011). Anaemia is the most prominent pathological feature of AAT (Taylor and Authié, 2004) and, in conjunction with other systemic lesions, can contribute to death through eventual congestive heart failure. Other symptoms include pyrexia, lymph node and spleen enlargement, ataxia, lethargy, weight loss, oedema, immunosuppression, abortion and decrease in milk production. The immunosuppression caused by trypanosomes can affect animal health by interfering with vaccination against other diseases (Singla *et al.* 2010), or by increasing susceptibility of the host to other infections. Inflammatory, degenerative lesions are also observed, and can damage various organs such as heart, central nervous system (CNS), eyes, testes, ovary and pituitary gland. Death may occur within weeks from onset of the acute disease. Otherwise the animal enters a chronic phase (spontaneous recovery is rare but not unknown), characterized by intermittent or sub-patent parasitaemia, general malaise and infertility, and may last months or years prior to death (Taylor and Authié, 2004).

While mortality due to the disease is clearly important, the impact upon overall cultivation and crop production due to reduced draught power is the most significant contributor to the economic impact of AAT (Swallow, 2000). This is considered the livestock disease with the highest impact on agricultural production and animal husbandry in Africa, causing annual losses which run to billions of US$ (Shaw *et al.* 2014). Across the tsetse belt as many as 55 million cattle are at risk of infection (Cecchi and Mattioli, 2009), plus 30 million sheep and 40 million goats. Of these cattle, 3 million die every year from AAT. The disease has devastating effects on the livelihoods of local farmers, for whom cattle represent not only a source of food (meat and milk), manure, and draught power, but have also fundamental social roles as ‘living banks’ and are used for social obligations (e.g. dowry and ritual use) (Swallow, 2000; Grace *et al.* 2009; Mungube *et al.* 2012).

Infection with *T. vivax* is considered an emerging disease in South America where it has a significant impact on cattle farming, but where it also affects horses and other ruminants (Batista *et al.* 2007, 2009, 2012; Da Silva *et al.* 2011). In a region including the Brazilian Pantanal and the Bolivian lowlands, where cattle ranching is the single most important economic activity (11 million head of cattle are reared in the region), the losses caused to the industry by a single outbreak of *T. vivax* in 1995 were calculated at more than US$ 160 million (Seidl *et al.* 1999). The gross financial burden of *T. vivax* in South America, however, is not known with any degree of certainty.

### Surra

Surra (from the Hindi word for ‘rotten’) is the most widely used of a plethora of names given to *T. b. evansi* infection in animals (Desquesnes *et al.* 2013*b*). As seen for *T. vivax, T. b. evansi* (a *T. brucei* subspecies) has also evolved a mechanical mechanism of transmission that has allowed this species to spread beyond Africa by export of infected animals (Lun *et al.* 2010). *Trypanosoma b. evansi* is today the pathogenic animal trypanosome with the broadest geographical distribution (Fig. 2), which stretches from North-East Africa to much of Asia in the east (Luckins, 1988; Payne *et al.* 1991; Lun *et al.* 1993) and to Latin America in the west (Desquesnes *et al.* 2013*b*), and it is spreading steadily. In Europe, recent imported cases of surra have been documented and vigilance remains necessary after outbreaks in the Canary Islands, mainland Spain, France and Germany (Desquesnes *et al.* 2008; Gutierrez *et al.* 2010; Tamarit *et al.* 2011; Defontis *et al.* 2012).

Several probable or suggested methods of surra transmission exist: by biting insects including horseflies and stable flies (the major credited route), by vampire bats, by iatrogenic (e.g. as a result of a vaccination intervention), sexual, horizontal or vertical transmission, or by per-oral contamination in the case of carnivores eating infected meat (Desquesnes *et al.* 2013*a*).

*Trypanosoma b. evansi* can parasitize a wide range of wild and domestic animal hosts, but the infection is particularly pathogenic in horses, camels and Asian water buffaloes (Desquesnes *et al.* 2013*b*). There is increasing evidence that common rodents are an important reservoir host for *T. b. evansi* and other trypanosomes (Jittapalapong *et al.* 2008; Maia da Silva *et al.* 2010; Kocher *et al.* 2015; Pumhom *et al.* 2015), such as *T. lewisi*, a parasite of rats also found in atypical human infections (Howie *et al.* 2006; Sarataphan *et al.* 2007). These findings revive the important question of rodents as reservoirs of other *T. brucei* species. Rare cases of human infection with *T. b. evansi* (Joshi *et al.* 2005; Haridy *et al.* 2011; Van Vinh *et al.* 2016), where individuals were infected through trypanosome-carrying animal blood, have been reported and, in at least one case, infection was associated with a null mutation in the trypanosome lytic factor blood component Apolipoprotein L1 (APOL1), which normally protects humans from animal trypanosome infections (Vanhollebeke *et al.* 2006; Truc *et al.* 2013). In a more recent case, no mutations in APOL1 were found to explain the unusual infection (Van Vinh *et al.* 2016).

Symptoms of surra overlap those previously described for AAT and their intensity can vary greatly between and within host species and depend on the geographical area and epidemiological situation (Desquesnes *et al.* 2013*b*).

In the Philippines, outbreaks of surra cause high morbidity and mortality in water buffaloes and other large ruminants, greatly affecting the livelihood of local small-scale farmers (Dargantes *et al.* 2009; Desquesnes *et al.* 2013*a*). In the Brazilian Pantanal *T. b. evansi* affects over 6000 horses per year (of the 50 000 present), with serious consequences to the local economy, horses being essential for herding livestock. The total impact of *T. b. evansi* infection in horses in this region was estimated at US$ 2·4 million per year (Seidl *et al.* 1998). Surra is also one of the most frequent diseases affecting camels in North Africa, causing severe economic damage.

### Dourine

Dourine is a disease caused by the subspecies *T. brucei equiperdum*, the only Salivarian trypanosome whose transmission cycle avoids invertebrate vectors completely. Instead, this parasite is transmitted among horses and other equids during mating (Claes *et al.* 2005). Of note, vertical or perinatal transmission of trypanosomes other than *T. b. equiperdum* in the reproductive tissues has been reported (Griffin, 1983; Melendez *et al.* 1993; Lindner and Priotto, 2010; Biteau *et al.* 2016), although the role and relative importance of this mode of transmission in the field is not clear.

*Trypanosoma b. equiperdum* is an important veterinary trypanosome endemic in Africa and Asia, and is also found in the Middle-East, South-East Europe and South America. Strict control policies have eradicated *T. b. equiperdum* from Western Europe in the past century (Claes *et al.* 2005), but the risk of reintroduction remains, as shown by a recent outbreak in Italy (Pascucci *et al.* 2013).

The infection presents with typical oedema of the genital organs as well as weakness, emaciation, urethral discharge, characteristic plaques in the skin and neurological symptoms such as lack of coordination of the hind legs (Hagos *et al.* 2010). Dourine in horses is generally fatal without treatment but it is usually subclinical in donkeys and mules (Brun *et al.* 1998).

Considering the transmission mechanism and the absence of a reservoir in other species, the control strategies for the disease follow a different approach as compared with other insect-borne forms of trypanosomiasis (Claes *et al.* 2005). The World Health Organization for Animal Health (OIE) recommends breeding and movement restrictions, compulsory notification and slaughter of infected animals to block new infection outbreaks or achieve eradication. Additionally, pharmacological therapy is not advised as this may result in clinical improvement but not in complete cure, leaving the animal as a potential carrier of the parasite. However, the feasibility or effectiveness of this strict policy in developing countries, where horses have a significant role in transport and agriculture, is questionable. Here, chemotherapy may help to sustain animal health and productivity. Although no official cure for dourine is available, studies have indicated the efficacy of melarsomine in the treatment of acute and chronic *T. b. equiperdum* infection in horses (Hagos *et al.* 2010).

## ANIMAL TRYPANOSOME SPECIES: VIRULENCE, TISSUE DISTIBUTION, BIOLOGY AND LABORATORY TOOLS

### *Trypanosoma congolense* and *T. simiae*

*Trypanosoma congolense* is the smallest of the pathogenic trypanosomes (see Fig. 1 for its morphology). The species is divided into three main subgroups (i.e. Savannah, Forest and Kilifi) based on molecular markers (Hide and Tait, 2004; Auty *et al.* 2015), the Savannah subgroup being the most virulent (Bengaly *et al.* 2002*a*, *b*) and the most clinically important in cattle. However, even within the same Savannah subgroup substantial differences in virulence exist, with some strains causing only mild infections (Masumu *et al.* 2006), highlighting the complexity and subtlety of the balance between the level of parasite persistence and the host immune system.

In the vertebrate host, *T. congolense* parasites remain confined to the vascular system, where they bind to circulating erythrocytes (Banks, 1979) and to endothelial cells (Hemphill *et al.* 1994) through their flagellum, causing damage at the adhesion site (Banks, 1980). Attachment of the bloodstream form is also observed in *in vitro* culture, where parasites adhere to the bottom of the flask, a phenotype unique to *T. congolense* among trypanosome species (Coustou *et al.* 2010).

Today, long-term culture of the pathogenic bloodstream form is possible only for a limited number of strains (e.g. IL3000 and STIB910) (Coustou *et al.* 2010). Genetic tools have been developed for this species, including a gene overexpression system (Coustou *et al.* 2010) and RNA interference (although, in this case, only for the procyclic insect form) (Inoue *et al.* 2002; Coustou *et al.* 2010). A draft genome sequence of strain IL3000 has also been published (Jackson *et al.* 2012) and offers the potential to accelerate discovery of biomarkers for diagnosis and targets for new drugs. However, despite the veterinary importance of *T. congolense*, the data available to understand its biology and pathogenicity and, therefore, to improve treatment, are scanty. It appears that this parasite has a carbohydrate metabolism that differs significantly from that of the far more widely studied *T. brucei* (Agosin and von Brand, 1954), with indications of a more pronounced mitochondrial activity in its bloodstream form. These dissimilarities may have relevance in the very different responses of these species to trypanocides (Leach and Roberts, 1981) and in the identification of potential drug targets. Of note, *T. congolense* lacks an orthologue of the *T. brucei TbAT1* gene that encodes the P2 nucleoside transporter (see subsection *Diminazene aceturate* below), which is central to the uptake of the trypanocidal drug diminazene (Munday *et al.* 2013). *Trypanosoma congolense* has a correspondingly reduced sensitivity to diminazene, which is not accumulated to the same degree in these parasites.

Similarly, the closely related *T. simiae* does not easily infect common laboratory rodents and, therefore, little data on this organism is available. However, a method for the axenic *in vitro* culture of the bloodstream form of this parasite has been published (Zweygarth *et al.* 1992), offering the means to accelerate our ability to dissect the parasite's biology.

### Trypanosoma vivax

**Fig. 3.**
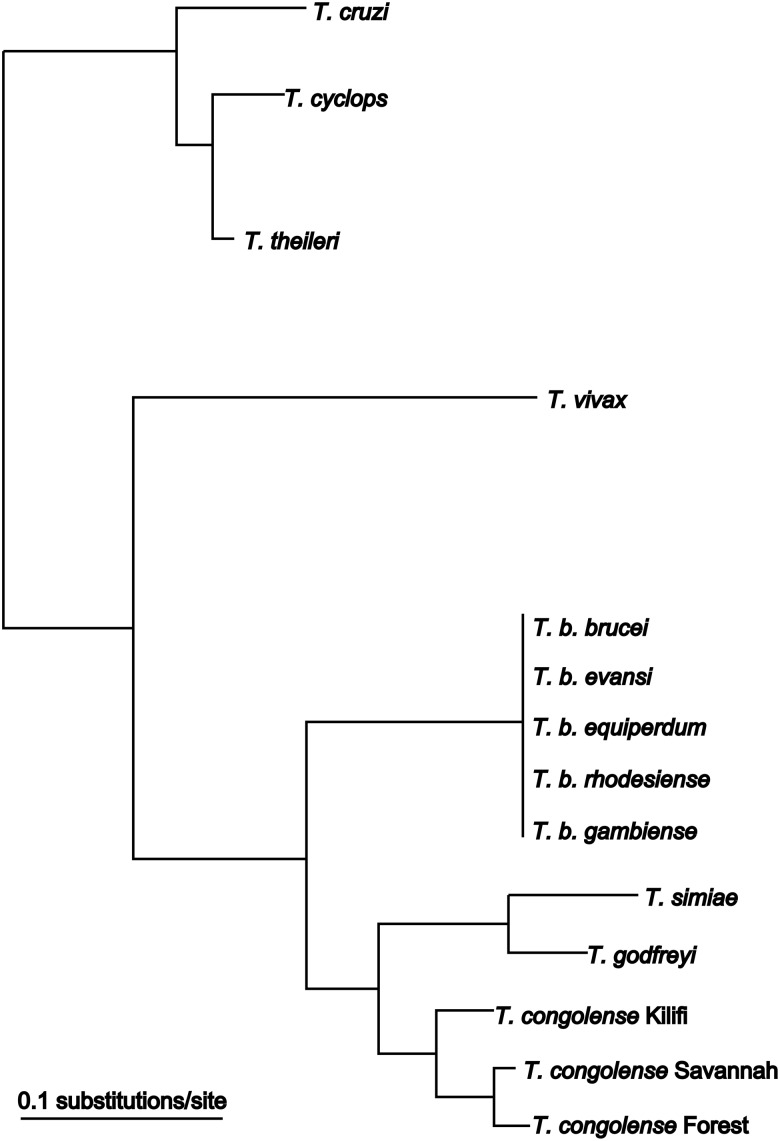
Phylogenetic tree based on SSU rRNA sequences from trypanosome species. Modified from (Cortez *et al.* 2006).

Among African trypanosomes, *T. vivax* (Fig. 1) is the most phylogenetically distinct species (Fig. 3). Specific isolates present with different pathogenicity in cattle, in some cases causing chronic, sub-clinical infections and in others acute, haemorrhagic infections (Wellde *et al.* 1983; Magona *et al.* 2008).

Although *T. vivax* (as *T. congolense*) has been considered typically to remain confined to the vascular system of the host, some strains may, especially in late infections, also reach extravascular locations (e.g. lymph nodes, eyes and cerebrospinal fluid) where they may directly damage tissues and where they are less accessible to drug treatment (Whitelaw *et al.* 1988; Osorio *et al.* 2008; D'Archivio *et al.* 2013).

*Trypanosoma vivax* is generally difficult to cultivate in the laboratory and this has restricted biological studies into this parasite. Short-term, axenic culture systems for the bloodstream form have been reported (Brun and Moloo, 1982; Zweygarth *et al.* 1991; D'Archivio *et al.* 2011) but they have been difficult to reproduce in other laboratories and have not entered routine use. Most studies on this trypanosome species are, therefore, conducted in *in vivo* laboratory models; however, very few *T. vivax* strains have been isolated that readily infect rodents and most published *in vivo* work on this species comprises the very few mouse-infective strains, the main one being Y486 and its derivatives (Gibson, 2012). A simplified system for *in vitro* cultivation of the insect form of *T. vivax* was recently described and genetic manipulation methodology implemented (D'Archivio *et al.* 2011). As with *T. congolense*, studies into the biochemical physiology of *T. vivax* have lagged behind those in *T. brucei* but significant differences with the metabolism of bloodstream form *T. brucei* were clear from early studies (Desowitz, 1956), which probably explains incongruence in potency of different chemical classes against these species.

### Trypanosoma brucei spp.

*Trypanosoma brucei* spp. (Fig. 1) include both animal (*T. b. brucei, T. b. evansi, T. b. equiperdum*) and human (*T. b. rhodesiense, T. b. gambiense*) infective subspecies. Unlike *T. vivax* (most strains at least) or *T. congolense, T. brucei* group trypanosomes are found in both the vascular system and in other tissues, and can parasitize the brain in experimental infections (Moulton, 1986; Grab and Kennedy, 2008; Coles *et al.* 2015); descriptions of this clinical condition in field settings are limited, other than for equids, which are particularly susceptible to *T. brucei* (Tuntasuvan *et al.* 1997; Ranjithkumar *et al.* 2014). As the most widely used drugs to treat animal trypanosomes (diminazene and isometamidium) do not cross the blood–brain barrier, the presence of parasites in sites other than the bloodstream represents a potentially important issue for treatment of *T. brucei*. Parasites from inaccessible body sites including the CNS may eventually re-establish infection in the bloodstream and cause relapse following treatment with these drugs (Myburgh *et al.* 2013). *Trypanosoma b. equiperdum* is quite unique, it being mainly a tissue parasite, found in the capillaries of the urogenital tract and rarely in peripheral blood (Brun *et al.* 1998). This makes diagnosis, parasite isolation and treatment particularly difficult.

*Trypanosoma b. brucei* is the most extensively studied trypanosome. Some lineages (e.g. Lister 427) are well adapted to laboratory *in vitro* culture and have been used as model organism to study many eukaryotic cell processes. The genome of this species was published in 2005 (Berriman *et al.* 2005) and its metabolism has been widely studied (Shameer *et al.* 2015). It has long been known that, in its bloodstream form, *T. brucei* species depend entirely on glycolysis for energy production, while Krebs cycle and oxidative phosphorylation are active only in the insect stages. New, comprehensive metabolomics approaches (Creek *et al.* 2015) are modifying this paradigm and, in conjunction with transcriptomic approaches, a clearer understanding of trypanosome metabolism is emerging.

*Trypanosoma b. evansi* and *T. b. equiperdum* can be considered *petite* mutants of *T. brucei*, so named after *petite* mutants of yeast that have lost mitochondrial respiratory function. These parasites have lost part (dyskinetoplastic parasites) or all (akinetoplastic parasites) of their kinetoplast DNA (kDNA), which constitutes the mitochondrial genome and comprises a network of circular concatenated mini- and maxi-circles (Schnaufer *et al.* 2002; Lai *et al.* 2008). Although long considered as two separate species, it has been proposed that *T. b. evansi* and *T. b. equiperdum* be reclassified as subspecies of *T. brucei*, based on phylogenetic analysis of sequenced genomes (Carnes *et al.* 2015), and we have adopted this convention here. As the kinetoplast genome encodes for an essential subunit (F_0_-A6) of the mitochondrial F_1_F_0_ ATP synthase, *T. b. evansi* and *T. b. equiperdum* cannot complete their life cycle in the fly and are locked in the trypomastigote stage, which relies on glycolysis for ATP production. A compensating mutation in the nuclear genome-encoded *γ*-subunit of the ATP synthase allows these parasites to maintain their mitochondrial membrane potential irrespective of the F_0_-A6 subunit and, therefore, to survive in the absence of the kinetoplast genome (Dean *et al.* 2013). It is for this reason that these parasites lost their dependency on the tsetse fly for transmission.

## CONTROL STRATEGIES AND TRYPANOTOLERANCE

All of the important livestock trypanosomes described above are extracellular parasites in mammals and evade the host immune defences by continuously changing their surface coat (Horn, 2014), one of the immune-evading mechanisms that essentially preclude the development of conventional vaccines (La Greca and Magez, 2011; Cnops *et al.* 2015). Hence, control of animal trypanosomiases relies primarily on the use of insecticides or traps to control the vector (especially in the case of tsetse-transmitted trypanosomiases), and on the use of trypanocides to control the parasite (Holmes, 2013). (The control strategy for dourine follows a completely different approach and has been described separately; see subsection *Dourine* above). Since vector control can be expensive when used on a large scale and is not always sustainable or effective, administration of trypanocidal drugs represents the main intervention tool in most poor rural endemic areas, ensuring maximum effects at relatively little cost (Grace *et al.* 2009; Van den Bossche and Delespaux, 2011). The cost-effectiveness of this practice was shown both in Africa (at least under certain circumstances) (Shaw *et al.* 2015) and elsewhere (Seidl *et al.* 1998, 1999; Dobson *et al.* 2009). Control of parasites with chemotherapeutic and chemoprophylactic agents has the double effect of limiting the losses caused by the infection and of eliminating the transmissible trypanosome reservoir (Welburn *et al.* 2015). Effective treatment of the acute phase of infection usually leads to prompt recovery of the animal; the use of trypanocides in the chronic phase, however, usually clears parasitaemia, but clinical recovery in these instances may require a significantly longer time, depending on the severity of symptoms such as weight loss and organ damage.

Some indigenous African livestock breeds (e.g. N'Dama, Muturu and Dahomey) are more resistant to trypanosome infection than imported breeds (classically temperate ‘European’ taurine breeds but also including Asian-derived *Bos indicus* breeds, relatively new to trypanosome endemic areas, such as Boran). This phenomenon is called ‘trypanotolerance’ and is defined as the ‘capacity to survive and remain productive after trypanosome infection’ (Murray *et al.* 1982). A major factor enabling these animals to cope with trypanosome infections is a better capacity to limit both anaemia and parasitaemia (Naessens, 2006). The use of trypanotolerant breeds has helped livestock productivity in various endemic regions in Africa and elsewhere, and it is often advocated as an important control strategy. Wild animals, which have co-evolved with trypanosomes, are also usually trypanotolerant and rarely suffer from clinical disease when infected.

## TREATMENT STRATEGIES AND CHALLENGES

Treatment and prophylaxis of pathogenic trypanosome infections in animals relies on only six compounds (Table 1), most dating back to the first half of the 20th century (Leach and Roberts, 1981). Moreover, several factors limit their use. The current drugs all have small therapeutic indices and can also cause local irritancy at the injection site. Most importantly, extensive utilization in the past has led to the appearance of resistant parasites in the field, and the fact that many of these trypanocides are chemically related has exacerbated the situation with cross-resistance onset (Peregrine, 1994). A number of currently used compounds appear to target the kinetoplast, causing its loss (Shapiro and Englund, 1990; Chitambo and Arakawa, 1992*b*), but the actual mode of action of these trypanocides and the biochemical mechanisms underpinning resistance are largely unclear. As noted above, differences in biochemical physiology and host organ distribution discriminate each of the veterinary trypanosomes and, therefore, the different trypanocides have divergent ability to kill based on specific potency against each species and pharmacokinetic parameters affecting distribution.

Most trypanocides have therapeutic rather than prophylactic activity, but the phenanthridine isometamidium is mostly used for its prophylactic effects (Stevenson *et al.* 1995). Unfortunately, these drugs are less active against *T. b. evansi* (Toro *et al.* 1983) and are less used outside of sub-Saharan Africa (Reid, 2002). The decision as to whether to use therapeutic or prophylactic drugs depends on several factors, including the risk of infection, drug availability and distribution logistics (Gu *et al.* 1999). Ideally, in areas of low prevalence, only those animals that present with clinical disease attributable to trypanosomes and/or have confirmed infection should be treated with therapeutic drugs; instead, in areas of high challenge, prophylactic drugs applied to the whole herd are more cost-effective, providing much greater reduction of mortality and morbidity and avoiding the adverse effects of infection on productivity (Gu *et al.* 1999). Single-dose therapeutic and prophylactic products for cattle are preferred, as multiple-dose administration regimens are often not practical in developing countries, where animal handling facilities are typically very limited.

As new compounds are not likely to become available in the near future (i.e. the most optimistic outlook is at least 3–5 years before a new compound could realistically be expected to be registered through current initiatives), prudent use of those already on the market is paramount. However, in field settings drug usage is often difficult to monitor and regulate. In hyperendemic African countries, trypanocides are usually administered directly by farmers, who can easily obtain them at local markets for a relatively affordable price (for less than US$ 1 per treatment). Unfortunately, most livestock keepers in the affected regions have limited access to tools which (a) enable accurate diagnosis, and frequently farmers are reliant solely on clinical signs, which are often not pathognomonic; and (b) provide information or training regarding optimal drug usage and dosage, and this combination of factors can lead to drug misuse (Van den Bossche *et al.* 2000; Grace *et al.* 2009). Moreover, in an unregulated market, poor quality or counterfeit trypanocides are widespread in some areas, especially in Africa, where documented product specifications are scarce (Sutcliffe *et al.* 2014; Tchamdja *et al.* 2016). To improve veterinary drug standards and tackle the issue of counterfeit drugs two laboratories for trypanocide quality control checks were recently set up in Africa (one in Dakar and one in Dar Es Salaam) thanks to a GALVmed-FAO (Food and Agriculture Organization of the United Nations) initiative with other collaborating partners (Sutcliffe *et al.* 2014).

Besides correct dosage administration, various other options to extend the life of current trypanocides exist. Different approaches (such as delivery systems including complexing to polymeric substances promoting slow release or alternative formulations) have been considered in order to improve therapeutic efficacy (Peregrine, 1994; Geerts *et al.* 1999; Kroubi *et al.* 2011; Unciti-Broceta *et al.* 2015). These could allow the use of lower quantities of trypanocide in a more effective way and, consequently, pose a decreased risk of toxicity and possibly decreased resistance development.

Unlike the situation with HAT, where the nifurtimox–eflornithine combination therapy (NECT) is now the preferred first line treatment for second-stage disease (Priotto *et al.* 2009; Alirol *et al.* 2013), no drug combinations are currently used for the animal trypanosomiases. Instead, alternating use of compounds, particularly diminazene and isometamidium (called a ‘sanative pair’), with low risk of cross-resistance, is recommended where possible. In particular, in the case of relapse the animal should be treated with a different drug class from the one previously administered, in order not to reinforce drug resistance selection (Leach and Roberts, 1981). Due to the chemical relatedness of several veterinary trypanocides, however, this approach is not always practicable. Thus, in order to maintain the efficacy of the currently used compounds, it is important that chemotherapeutic and chemoprophylactic dosage regimens are rationalized on the basis of the drug-susceptibility phenotype of trypanosome populations in a given locality. However, such rationalization is not possible, because the systems that are currently available to characterize the drug resistance phenotype of trypanosome populations are not field applicable (Peregrine, 1994). Limited numbers of field isolates can be characterized and all of the systems take many months to provide definitive data (see section *Tests for resistance detection* below). There is therefore a requirement for new assays that will rapidly quantify the drug resistance phenotype of large numbers of trypanosome isolates.

## VETERINARY TRYPANOCIDES: DOSAGE, PHARMACOKINETICS, MODE OF ACTION AND RESISTANCE

### Diminazene aceturate

Diminazene aceturate (Table 1) was introduced for the treatment of babesiosis and African trypanosomiasis in livestock in 1955. It belongs to the diamidine class of compounds, a member of which (pentamidine) has also been used for HAT since the 1930s (Steverding, 2010). Ironically, it was pursuing a structure-activity iterative synthesis from a compound belonging to a different class, Surfen C [at the time of its introduction in the 1930s, the best available agent against *T. congolense* infections (Bennett, 1936)], that led to diminazene development (Hawking, 1963). Although it was anti-*T. congolense* activity in experimental rodents that initially drove development, today's *in vitro* systems, where anti-parasite potency can be tested without confounding issues related to pharmacokinetic behaviour in hosts, show that diminazene is substantially less potent against *T. congolense* than it is against *T. brucei* group trypanosomes. This feature is attributable to the fact that its uptake into the latter parasites via the P2/*Tb*AT1 transporter (see later) allows concentrative and rapid uptake (De Koning *et al.* 2004). In *T. congolense*, which lacks an orthologue of *TbAT1* (Munday *et al.* 2013), uptake is less robust, explaining its lower activity.

Diminazene is today the most commonly used trypanocide in cattle, sheep and goats, due to its activity against both *T. congolense* and *T. vivax* and its relatively low toxic side effects. The compound also effectively cures surra and is, for example, the mainstay of treatment of *T. b. evansi* in the Philippines (Reid, 2002). The recommended therapeutic dose is 3·5 mg kg^−1^ body weight for AAT due to *T. congolense* and *T. vivax* (7 mg kg^−1^ may be recommended against resistant isolates) and 7 mg kg^−1^ is indicated for AAT due to *T. brucei* and for surra, administered by intramuscular or subcutaneous injection (Connor, 1992). The common practice of administering 3·5 mg kg^−1^ of the drug to treat *T. b. evansi* infections is considered an underdosing, and this misuse may have contributed to the emergence of resistant strains in South-East Asia (Desquesnes *et al.* 2013*a*). The fact that higher doses appear to be needed to treat *T. brucei* group trypanosomes, in spite of these parasites being more sensitive to the drug, probably relates to their wider tissue dispersal compared with *T. congolense* and *T. vivax*, underlining the key role of host pharmacokinetics.

Diminazene is only applied as a curative agent and is not used for prophylaxis, as it is rapidly metabolized and excreted (Peregrine and Mamman, 1993). After rapid absorption (the peak blood level is reached within 1 h of dosing), elimination follows a biphasic or triphasic behaviour depending on the animal species and formulation; elimination half-life values following intramuscular administration varied from 11–19 h in sheep and goats, to 74 to >200 h in cattle (Mamman *et al.* 1993; Peregrine and Mamman, 1993; Mdachi *et al.* 1995; El Banna *et al.* 1999). Cattle excrete diminazene mainly in the urine, together with two main metabolites: *p-*aminobenzamidine and *p-*amino-benzamide (Kellner *et al.* 1985). Diminazene residues may persist for several weeks in the edible tissues of cattle and other food-producing animals, especially in the liver and kidney, whereas the drug levels in milk peak at 6 h and fall to below detection limits after 48 h (FAO, 1990). For this reason it is advised that cattle and sheep destined for human consumption are subject to a 21–35 days pre-slaughter withdrawal (discard) from drug, while a 3-day milk discard period is recommended (FAO, 1990; Peregrine and Mamman, 1993); however, product-specific withdrawal periods as given on product labels should be adhered to.

The trypanocidal mode of action of diminazene has not been completely elucidated. The compound binds the minor groove of the DNA at AT-rich sites (Wilson *et al.* 2008). In trypanosomes, the kDNA is a known target of the drug, and kDNA binding can cause inhibition of replication and kDNA loss (Shapiro and Englund, 1990), possibly exacerbated by an inhibitory effect on mitochondrial type II topoisomerase (Portugal, 1994). It had long been believed that loss of the kinetoplast might not be sufficient to kill trypanosomes, as viable dyskinetoplastic strains do occur naturally and also can be produced artificially in the laboratory (Schnaufer *et al.* 2002). However, the discovery in laboratory generated-dyskinetoplastic *T. b. brucei* of a compensating mutation in the nuclear genome-encoded γ-subunit of the mitochondrial ATP synthase (Dean *et al.* 2013) meant that the kinetoplast has been resurrected as the potential drug target of diminazene. These dyskinetoplastic lines do indeed show significant *in vitro* resistance to diamidines (including diminazene aceturate) and phenanthridines (Gould and Schnaufer, 2014). Furamidine (DB75), a closely related diamidine, whose fluorescent properties enabled tracking of its cellular distribution, was shown to bind to *T. b. brucei* kDNA and nuclear DNA *in situ*, and also to accumulate in other organelles identified as acidocalcisomes (Mathis *et al.* 2006). The compound was also shown to interfere with the mitochondrial membrane potential (Lanteri *et al.* 2008). Interestingly, it has been suggested that diminazene can also modulate the host immune response by dampening pro-inflammatory cytokines and excessive immune activation, which might also influence the *in vivo* effects of the drug (Kuriakose *et al.* 2012).

Chemically, diminazene is an aromatic diamidine made of two benzamidine moieties linked by a triazene bridge. Due to its charged nature, diminazene can only cross membranes via specific carriers and this has three important consequences: (a) the drug is not active on CNS infections as it cannot cross the blood–brain barrier; (b) the compound is selectively toxic to trypanosomes, as they express transporters that specifically accumulate diminazene; and (c) trypanosomes may become resistant to the drug by losing these transporters or their activity. As mentioned above, diminazene uptake in *T. brucei* mainly occurs via an aminopurine transporter called P2 or *Tb*AT1, which is also implicated in the uptake of the related diamidine pentamidine and the melaminophenyl arsenical melarsoprol, two drugs licensed for HAT (Carter *et al.* 1995; Barrett and Fairlamb, 1999; De Koning, 2008). Diminazene uptake into *T. brucei* is fast, with a *Km* of 0·45 *µ*m and a *V*_max_ of 0·049 pm 10^7^ cells^−1^ s^−1^ (De Koning *et al.* 2004) and is inhibited by pentamidine and adenosine, the main physiological substrate of this carrier. Loss of P2/*Tb*AT1 activity was shown to cause diminazene resistance in *T. b. brucei* (Matovu *et al.* 2003), *T. b. equiperdum* (Barrett *et al.* 1995; Stewart *et al.* 2010) and *T. b. evansi* (Witola *et al.* 2004). Another gene, named *TeDR40*, has also been implicated in resistance in *T. b. evansi* (Witola *et al.* 2005). However, using that gene to search for orthologues in other trypanosomatids at the TriTrypDB database (www.tritrypdb.org), indicates that it is actually a variant surface glycoprotein (VSG) gene, part of the parasite's system of antigenic variation whereby it avoids host immunity. It is possible that, in the process of selection of resistance, the parasites switched expression of a VSG gene independently of the resistance selection, which explains the massive increase in expression of that gene.

The application to *T. brucei* of a genome-wide RNA interference target sequencing (RIT-seq) screen, where any gene whose loss of function is identified by reduced drug sensitivity, was able to identify additional plasma membrane proteins (P-type H^+^-ATPases), as well as a putative protein phosphatase, that were linked to the action of the related diamidine pentamidine (Alsford *et al.* 2012). The HAPT1/*Tb*AQP2 carrier (De Koning, 2001*b*), encoded by the *TbAQP2* gene (Baker *et al.* 2012), has a key role in uptake of pentamidine and the melaminophenyl arsenicals in *T. brucei*, although its role in diminazene uptake is less pronounced (Teka *et al.* 2011; Munday *et al.* 2014) and loss of P2/*Tb*AT1 alone is sufficient to give high level of resistance to this latter drug (Matovu *et al.* 2003). It has recently been proposed that *Tb*AQP2 acts as a receptor for pentamidine, with high affinity, and its uptake then occurs via receptor-mediated endocytosis (Song *et al.* 2016); further work is needed to confirm or refute this hypothesis, although other evidence points to pentamidine actually entering through the channel, enabled by a unique selectivity filter and the high degree of flexibility of the pentamidine chain (Munday *et al.* 2014, 2015*a*).

*Trypanosoma congolense* appears to lack a functional equivalent of *Tb*AQP2. A putative P2/*Tb*AT1-type transporter, *Tco*AT1, was identified in *T. congolense* and a particular allele proposed to be associated with diminazene resistance (Delespaux *et al.* 2006). This conclusion was curious, given that the so-called resistance allele was not always associated with resistant form parasites isolated in one region (Delespaux *et al.* 2006) and was also abundant in areas where diminazene had not been used (Chitanga *et al.* 2011). Furthermore, *TcoAT1* is not the orthologue of *TbAT1*, instead corresponding to a related, but distinct, member of the nucleoside transporter family (Munday *et al*. 2013). Its heterologous expression has proven that the encoded protein does not enable diminazene uptake, instead facilitating the uptake of adenosine and inosine (Munday *et al.* 2013). Hence, it can be definitively ruled out that the gene misnamed *TcoAT1* has any role in diminazene uptake, action, or resistance.

Diminazene resistance is generally believed to be difficult to produce experimentally in *T. congolense* (in contrast to *T. brucei*). High levels of resistance to the drug were obtained in mice infected with *T. b. evansi*, but only when using immunocompromised animals, a result which stresses the importance of the link between immunity and chemotherapy, as the efficacy of trypanocides appears to be reduced by immunosuppression, hence favouring development of resistance (Osman *et al.* 1992). *In vitro* experiments with *T. b. brucei* and *T. b. evansi* demonstrated that a shared mechanism of internalization accounts for the cross-resistance between diminazene and other diamidines as well as melaminophenyl arsenicals (melarsoprol and melarsomine) (Matovu *et al.* 2003). By contrast, no cross-resistance was observed with other chemically unrelated compounds including suramin or quinapyramine. A degree of cross-resistance has been observed between isometamidium and diminazene in *T. brucei* group trypanosomes, although the functional basis of this is not clear (Zhang *et al.* 1991; Witola *et al.* 2004).

### Homidium salts

Homidium bromide or ethidium bromide, also available as a chloride salt (Novidium^®^, Table 1), was introduced for field use in 1952, as an improvement to previous phenanthridine-based trypanocidal agents (Wainwright, 2010). It is widely used in Africa to treat *T. congolense* and *T. vivax* infections in cattle, sheep and goats, in spite of its proven mutagenic and possible carcinogenic properties as a DNA intercalator (Sutcliffe *et al.* 2014). Due to its potential toxicity, the use of homidium is today highly discouraged (Sutcliffe *et al.* 2014). Widespread resistance to the drug in the 1960s and 1970s reduced its usage. Today, the number of doses of homidium used annually is reported to be down to around 10% of the total African trypanocide market, but this value may be a significant underestimate of its real use (Frans van Gool, personal communication, 2015).

Although used as a curative drug, homidium also possesses chemoprophylactic properties, but these are less pronounced than those of isometamidium (see subsection *Isometamidium chloride* below). For both purposes, homidium is administered at the dose of 1 mg kg^−1^ by a single, deep intramuscular injection (Peregrine, 1994). Homidium excretion is faster than isometamidium, its serum concentration declining rapidly over the first 24 h following both intravenous and intramuscular injection at a standard dosage (Murilla *et al.* 1999). Elimination half-life ranged from 178 h in Boran cattle to 488 h in Friesian cattle following intramuscular injection (Murilla *et al.* 1999). However, low levels of the drug (0·1–0·3 ng mL^−1^) do persist in circulation for several weeks when given intramuscularly, providing an 8–17-week prophylaxis period (Dolan *et al.* 1990; Murilla *et al.* 1999). Homidium has an extensive extravascular distribution and accumulates predominantly in the liver and the kidneys (Murilla *et al.* 1996), a factor which presents some risk in products from treated animals destined for human consumption. Homidium can be used as sanative pair with diminazene, but not with isometamidium, where the shared phenanthridine core underlies cross-resistance (Peregrine *et al.* 1997).

Intracellular localization of homidium can be monitored by microscopy, exploiting the intrinsic fluorescence of the compound. Work on *T. brucei* showed that homidium localizes in the nucleus and the kinetoplast of treated trypanosomes (Cox *et al.* 1984; Boibessot *et al.* 2002). Treatment with the drug induces dyskinetoplasty in a similar way to other phenanthridines and diamidines (Riou *et al.* 1980; Shapiro and Englund, 1990) and disruption of genome function has long been believed to underlie its trypanocidal effects. Indeed, it was found that homidium blocks both kinetoplast and nuclear DNA replication in *T. brucei* by distorting and changing the double helix topology (Roy Chowdhury *et al.* 2010). The inhibition of minicircle replication and, consequently, loss of the kinetoplast network, was found to be the primary killing mechanism at low doses (0·02 *µ*g mL^−1^), but at higher doses homidium was also shown to affect nuclear DNA, which could account for its ability to kill dyskinetoplastic trypanosomes (Roy Chowdhury *et al.* 2010). The reason for the initial targeting of the kinetoplast over the nucleus is believed to be the result of the preferential accumulation of lipophilic cations (such as homidium) in the mitochondrion, as shown with other experimental trypanocides (Lanteri *et al.* 2008; Ibrahim *et al.* 2011; Alkhaldi *et al.* 2016). The mechanism of resistance to homidium is not known, but it is likely to be similar to that of the related compound isometamidium.

### Isometamidium chloride

Isometamidium chloride hydrochloride is a hybrid phenanthridine with amphiphilic and cationic properties, synthesized by coupling homidium with the diazotized *p-*aminobenzamide moiety of diminazene, modified with the amidine group in the *meta* position (see Table 1 for structures). It has both curative and prophylactic properties and, since its launch in the 1960s, it has remained the only drug available for chemoprophylaxis of AAT, after quinapyramine was discontinued due to problems linked to toxicity and, particularly, the induction of multi-drug resistance (Peregrine, 1994; Geerts and Holmes, 1998). The veterinary formulations are typically a mixture of four phenanthridine compounds: isometamidium chloride hydrochloride [8-(3-*m*-amidinophenyl-2-triazeno)-3-amino-5-ethyl-6-phenylphenanthridinium chloride hydrochloride], the positional red isomer [3-(3-*m*-amidinophenyl-2-triazeno)-8-amino-5-ethyl-6-phenylphenanthridinium chloride hydrochloride], the blue isomer [7-(*m*-amidinophenyldiazo)-3,8-diamino-5-ethyl-6-phenylphenanthridinium chloride hydrochloride], and the disubstituted compound [3,8-di(3-*m*-amidinophenyltriazeno)-5-ethyl-6-phenylphenanthridinium chloride dihydrochloride]. A protocol for their individual purification from the mixture and a detailed structural analysis of each compound were described in a recent publication (Igoli *et al.* 2015). In commercial products isometamidium is the principal component (guidelines establish it must be at least 55% of the total material), with the other components accounting for less than 40% (Sutcliffe *et al.* 2014). As the *in vitro* and *in vivo* trypanocidal activity on *T. congolense* is lower for the red and blue isomer it is paramount that the product composition follows strict quality standards (Sahin *et al.* 2014). The disubstituted compound has poor trypanocidal activity but it has a good prophylactic effect, possibly because it can act as a pro-drug that is cleaved to isometamidium *in vivo* (Sahin *et al.* 2014).

Isometamidium is used primarily to treat and prevent *T. congolense* and *T. vivax* infections in livestock in Africa. Its activity against *T. brucei* spp. is less marked, but this drug can also be utilized against some *T. b. evansi* strains, although not when these have reached the CNS, as the compound does not cross the blood–brain barrier. The drug is administered to cattle at single doses of 0·25–1·0 mg kg^−1^ for cure, and at doses of 0·5–1 mg kg^−1^ for prophylaxis (Leach and Roberts, 1981). The dosage for *T. b. evansi* infections is generally 1–2 mg kg^−1^, but in horses it is recommended not to exceed 0·5 mg kg^−1^ due to toxicity issues (Uilenberg, 1998; Desquesnes *et al.* 2013*a*). Multiple intramuscular administrations of isometamidium can cause severe fibrous lesions, hence damaging the carcass and meat quality from livestock. Intravenous administration has been successfully used to abrogate muscular damage, but it has been suggested that this could result in compromised prophylactic activity, due to the lack of a drug depot at the injection site (Dowler *et al.* 1989; Munstermann *et al.* 1992). The duration of prophylactic activity following intramuscular administration in cattle is typically 2–3 months and may be up to 6 months, but can vary greatly, depending on the formulation and dosage used and on the parasite strain, as well as on other factors, including susceptibility of the particular breed and its general health status (Toro *et al.* 1983; Kinabo and Bogan, 1988).

Isometamidium plasma concentrations reach their peak within 1 h after administration and then fall relatively quickly during the first week post-treatment and thereafter more gradually (Kinabo, 1993; Eisler *et al.* 1994). Three months after cattle had been injected, the circulating drug concentration was measured at 0·75 ng mL^−1^ (Eisler *et al.* 1994). This study showed that the serum concentration fits a bi-exponential model, with half-life of approximately 25 days for the second phase in cattle (Eisler *et al.* 1994), while another study (Eisler, 1996) indicated an elimination half-life of 9–19 days. In sheep and goats isometamidium appears to be eliminated more rapidly than in cattle (Wesongah *et al.* 2004). The drug accumulates in the liver, kidneys and spleen as well as at the injection site, and from here it is slowly released to the plasma exerting its prophylactic activity (Kinabo and Bogan, 1988). Persistence of isometamidium residues is much longer than for diminazene. For this reason, a withdrawal period of 30 days was established for consumption of produce from cattle treated with the drug (FAO, 1990), although in practice the withdrawal (discard) period is always product-specific. Excretion occurs mainly via bile and levels in cattle milk are generally very low (Kinabo, 1993).

Isometamidium may be used as part of a sanative pair with diminazene, the two drugs being used sequentially to minimize the risk of resistance development (Leach and Roberts, 1981; Peregrine, 1994). Despite this recommendation, there are multiple reports of field isolates, from many African countries, indicating isometamidium resistance, particularly in *T. congolense* but also in *T. brucei* species and *T. vivax*, sometimes detailing cross-resistance with diminazene (Ainanshe *et al.* 1992; Clausen *et al.* 1992; Codjia *et al.* 1993; Afewerk *et al.* 2000; Sinyangwe *et al.* 2004; Mamoudou *et al.* 2008). However, other reports found no cross-resistance (e.g. Gray *et al.* 1993; Joshua *et al.* 1995) and we conclude that cross-resistance does not necessarily occur, but may be a consequence of the level of resistance that has been established, whereas in other cases resistance to both drugs may have been induced separately. In addition, the chance of cross-resistance developing may be different for the various animal trypanosome species, given their known differences in biochemical physiology and drug transport.

By taking advantage of isometamidium's intrinsic fluorescence, accumulation in the kinetoplast was observed (Wilkes *et al.* 1995; Boibessot *et al.* 2002). Although closely related to the intercalating phenanthridine homidium, isometamidium is not known to be carcinogenic, and was reported to bind kDNA with an unconventional ‘sideways’ geometry (Dougherty and Waring, 1982). Its high affinity for the kDNA might underlie its trypanocidal activity. Linearization of kDNA minicircles in *T. b. equiperdum* following interaction of the drug with the kinetoplast was observed (Shapiro and Englund, 1990). Moreover, naturally occurring dyskinetoplastic *T. b. evansi* (Brun and Lun, 1994) and *in vitro*-generated *T. b. brucei* lacking a functional kinetoplast (Gould and Schnaufer, 2014) are highly resistant to the drug. Efficacy against some *T. b. evansi* strains might relate to these parasites retaining kDNA (albeit dispersed in dyskinetoplastidy) while others are akinetoplastic (i.e. retain no kDNA at all) and may be less susceptible to the drug. However, the drug would still accumulate preferentially in the mitochondrion, as the mitochondrial membrane potential is unaffected by the loss of the kinetoplast in cells carrying a compensatory mutation in the *γ*-subunit of the F_1_F_0_-ATP synthase (Dean *et al.* 2013), providing a driving force for cations. A mutation in this ATP synthase subunit is sufficient to cause a substantial level of isometamidium and homidium resistance, although further drug pressure was shown to increase this even further. Interestingly, this very high level of resistance is indeed associated with a loss of mitochondrial membrane potential, preventing further isometamidium accumulation in this organelle (Eze *et al.* 2016).

Despite possessing the recognition motif for the P2/*Tb*AT1 transporter and despite being a high-affinity inhibitor of this carrier (De Koning, 2001*a*), the internalization of isometamidium depends at most partially on this route (Delespaux and de Koning, 2007). Passive diffusion across the membrane may be feasible but is not likely, given the two positive charges on the molecule and partial characterization of isometamidium transport, linking drug resistance, at least in part, to reduced uptake (Sutherland *et al.* 1992; Wilkes *et al.* 1995, 1997). High-throughput RIT-seq (Baker *et al.* 2015) failed to identify involvement of any of the receptor-mediated endocytosis pathways as previously identified for suramin (see subsection *Suramin sodium* below) using this approach (Alsford *et al.* 2012), although alternative endocytic routes could not be ruled out.

Resistance to isometamidium is encountered in the field. In *T. congolense* a mechanism behind resistance was proposed to relate to diminished mitochondrial membrane potential (Wilkes *et al.* 1997). This, in turn, would diminish the accumulation of drug in the mitochondrion, having a net effect of reduced uptake at the plasma membrane, presumably due to rapid equilibration of intracellular and extracellular concentrations when the mitochondrial sink is lost. Active extrusion by plasma membrane transporters has also been proposed (Sutherland and Holmes, 1993). A recent application of the RIT-seq approach, conducted on *T. brucei*, identified mutations to many subunits of the vacuolar ATPase (found in the lysosomes and acidocalcisomes), in the trafficking protein AP-3 (an adaptin that mediates delivery of proteins to lysosome-related organelles) and in EMC (an ER membrane complex) that reduced drug activity, potentially contributing to dug resistance (Baker *et al.* 2015). Secondary loss of kDNA was found to be possible once vATPase and AP-3 subunits are lost from the cells, pointing to an intriguing, but as yet ill-defined, interaction between the vacuolar system and mitochondrion. The fact that kDNA is lost in cells selected for resistance to isometamidium was classically interpreted to point to its role as target. However, the discovery that kDNA loss can occur as a consequence of changes to the vacuolar system complicates this interpretation.

### Quinapyramine sulphate

Quinapyramine sulphate was developed from the early trypanocide Surfen C (Curd and Davey, 1950) and came into use around 1950. The compound was applied to treat cattle infected with trypanosomes until 1976, when it was withdrawn from many areas due to emergence of widespread resistance (Connor, 1992). The drug was subsequently reintroduced in 1984 to treat *T. b. evansi* in camels and horses (Peregrine, 1994), and is still used today (Ranjithkumar *et al.* 2014). In horses with acute infections of *T. brucei* spp. quinapyramine is considered the most effective treatment (5 mg kg^−1^ via subcutaneous injection), although the drug induces severe but transient side effects in these animals (Auty *et al.* 2008). The prosalt form of quinapyramine (a mixture of the soluble sulphate and the insoluble chloride salts) was the first prophylactic drug available for animal infections. A 7·4 mg kg^−1^ dose of this prosalt suspension has both a curative and a prophylactic (up to 4 months) effect on *T. b. evansi* infections in horses and camels (Williamson, 1970).

Quinapyramine is a quinoline pyrimidine (Table 1) and, as isometamidium and diminazene, a dication at physiological pH (homidium is monocationic). As seen for the other charged trypanocides, quinapyramine is unable to cross the blood-brain barrier, which explains its failure to cure *T. b. evansi* infections in equids when the CNS is affected (Ranjithkumar *et al.* 2014). However, it is important to note that some cationic trypanocides do penetrate the blood–brain barrier, the clearest example being compound DB829 (Wenzler *et al.* 2013). Pentamidine has actually been used to treat ‘early-late stage’ HAT (Doua *et al.* 1996) but its movement across the blood–brain barrier is counteracted by active efflux mechanisms, including P-glycoprotein and multi-drug resistance transporters (Sanderson *et al.* 2009).

Plasma levels of quinapyramine decline rapidly after dosing and, in the case of the prosalt, its persistence is probably due to slow release from the subcutaneous depot formed at the injection site (Spinks, 1950). Quinapyramine accumulates in the liver and kidneys, where its concentration remains high for weeks and can cause organ-specific toxicity. Excretion occurs mainly via urine (Spinks, 1950).

Quinapyramine's mode of action remains unknown. Hypotheses include the interference with nucleic acid synthesis and inhibition of cytoplasmic ribosomes (and, therefore, protein synthesis) (Newton, 1962, 1966). However, its dicationic/aromatic nature would strongly suggest a mitochondrial accumulation, as with the phenanthridines and bis-benzamidines.

*Trypanosoma congolense* and *T. b. evansi* lines resistant to the drug can easily be obtained by *in vivo* selection in mice (Ndoutamia *et al.* 1993; Liao and Shen, 2010). As quinapyramine resistant *T. congolense* trypanosomes show cross-resistance to isometamidium, homidium and diminazene, the use of this compound to treat infections in cattle is not recommended (Peregrine *et al.* 1997). Given the lack of cross-resistance between diminazene and homidium, the fact that quinapyramine is cross-resistant to both is intriguing. Although the mechanism underpinning quinapyramine resistance remains unknown, it is likely that all these trypanocides have a mitochondrial target and that any single change that dramatically reduces the mitochondrial membrane potential, or the loss of organic cation carriers in the inner mitochondrial membrane, could result in resistance to all of them.

### Suramin sodium

Suramin sodium is a symmetrical polyanionic sulfonated naphthylamine (Table 1). It is the oldest trypanocide still in use, having been introduced in 1921 for the treatment of surra in camels and replacing the then-standard treatment of intravenous tartar emetic (potassium antimonyl tartrate) (Uilenberg, 1998). A single dose of 6–10 g of suramin sodium per camel was described as 100% effective (Bennett, 1930). Suramin is also the standard treatment for equine trypanosomiasis (*T. brucei* spp.), being more effective than diminazene and less toxic than quinapyramine (Williamson, 1970). The current treatment for camels and horses is 10 mg kg^−1^, administered intravenously. Intramuscular administration is avoided as it causes intense local irritation. Suramin has further been used for cure and prophylaxis of onchocerciasis and other microfilarial infections including *Brugia pahangi* (Delespaux and de Koning, 2007), as well as for the treatment of early stage HAT since 1922 (Apted, 1970). Although suramin is effective against *T. b. gambiense* (Knobloch *et al.* 1984; Pepin and Khonde, 1996), it is mostly used against HAT due to *T. b. rhodesiense*, for which it is still available today (Voogd *et al.* 1993), whereas it was replaced with pentamidine for the form due to *T. b. gambiense*. Although the drug has good efficacy against *T. simiae* in pigs (Stephen, 1966; Williamson, 1970), it is relatively ineffective against *T. congolense* and *T. vivax* (Leach and Roberts, 1981), presumably due to the aforementioned differences in biochemical physiology that distinguish *T. brucei* group organisms from these other species.

Old work showed that suramin can be used as a prophylactic agent when administered subcutaneously as an insoluble complex with one of the cationic trypanocides (e.g. with quinapyramine, in a 1:3 molecular proportion, reflecting the six negative charges of suramin *vs* the two cationic charges of quinapyramine), resulting in 3–6 months protection at 40 mg kg^−1^ of quinapyramine in pigs (Williamson, 1970) and >160 days protection in cattle (Williamson and Desowitz, 1956). This approach could be effective for the eradication of *T. b. gambiense* in pigs, which are reportedly acting as reservoir hosts of this species (Mehlitz *et al*. 1982). Complexes of suramin with homidium, quinapyramine and prothidium also gave protection in experimental infections in cattle (Desowitz, 1957).

The pharmacokinetic parameters of suramin in animals (Kinabo, 1993) have not been subject to the same extensive characterization as occurred in humans, where the compound has also been trialled for the treatment of AIDS and cancer (Barrett *et al.* 2007). Most of the drug (>99%) binds to plasma proteins yielding a slow clearance. The terminal half-life in humans ranges between 40 and 50 days or more, depending on the infusion protocol applied (Jodrell *et al.* 1994). This slow clearance underpins limited (i.e. several weeks) prophylactic action in animals too when the drug is used on its own. Suramin does suppress infection, but is dependent on the host's immune response to be fully effective (Leach and Roberts, 1981). Because of its large molecular size and highly anionic nature, suramin does not cross the blood–brain barrier.

Suramin strongly binds to human serum proteins and various trypanosome enzymes by electrostatic interaction (Voogd *et al.* 1993). The drug was proposed to enter trypanosomes via receptor-mediated uptake bound to LDL and to accumulate in the lysosome (Vansterkenburg *et al.* 1993). This hypothesis, however, looked doubtful after it was demonstrated that in *T. brucei* (procyclic form at least) suramin and LDL uptake are not coupled (Pal *et al.* 2002). A definitive mode of action for the compound has not been determined. Fairlamb and Bowman proposed that suramin curbs glycolytic ATP production in *T. brucei* by inhibiting glycerol-3-phospate oxidase and NAD^+^-dependent glycerol-3-phosphate dehydrogenase (Fairlamb and Bowman, 1980). However, being highly charged, suramin binds many enzymes when assayed and a multitude of putative targets have been proposed (Gutteridge, 1985), including 6-phosphogluconate dehydrogenase, of the pentose phosphate pathway, of which it is a competitive inhibitor (Hanau *et al.* 1996). More recently, a RIT-seq screen in bloodstream *T. brucei* identified 28 genes that contribute to suramin action, including: a surface glycoprotein family (ISG75), which appears to be the ligand to which the drug binds; cathepsin L, believed to release the drug from ligand within the lysosomal system; a number of deubiquitinating enzymes and various proteins involved in the endocytic pathway (Alsford *et al.* 2012). It appears that inhibiting uptake of suramin, or its normal passage through the endocytic pathway following binding to a specific receptor, is sufficient to render parasites resistant to the drug, although it remains unknown how suramin kills once accumulated intracellularly.

Extensive use of the compound in the first half of the 20th century resulted in emergence of widespread resistance in *T. b. evansi* in Africa (Boid *et al.* 1989; El Rayah *et al.* 1999) and South-East Asia (Gill, 1971; Zhou *et al.* 2004), in some cases leading to withdrawal of suramin as a treatment (El Rayah *et al.* 1999). However, even in the absence of drug pressure, the resistance phenotype has persisted in the field, as found for some Sudanese *T. b. evansi* strains (El Rayah *et al.* 1999). Stability of the suramin resistance phenotype was also observed in *T. brucei* lines generated *in vitro* (Scott *et al.* 1996) and in *T. b. evansi* parasites selected in mice (Mutugi *et al.* 1994). However, the drug was effective against *T. b. evansi* isolates in Brazil, where it had not been used (Faccio *et al.* 2013).

### Melarsomine dihydrochloride

An early reported case of an attempt to cure an animal afflicted with trypanosomiasis was that of Dr David Livingstone, the Scottish missionary whose travels in Southern Africa in the mid-19th century were exceptionally well recorded. In a letter to the British Medical Journal in 1858 he described the use of arsenic oxide (Fowler's solution) to treat a case of ‘fly disease’ in a horse (Livingstone, 1858). Although the treated horse was not cured, there was a temporary relief in symptoms. Over 50 years later, once the trypanosome had been implicated, H. W. Thomas and A. Breinl, and then P. Ehrlich, revisited arsenic chemistry to seek trypanocides in the early days of chemotherapy (Williamson, 1970). By the 1950s melarsoprol had been introduced for the treatment of late-stage HAT; the drug was created by coupling of melarsen oxide to 2,3-dimercaptopropanol (Steverding, 2010). The formulation displayed diminished toxicity while retaining potent trypanocidal activity.

Melarsomine dihydrochloride (Table 1) is a melamino-phenylarsine, synthesized by linking melarsen oxide (Barrett *et al.* 2007) to two equivalent of cysteamine (Berger and Fairlamb, 1994). The compound has improved aqueous solubility over melarsoprol. It was introduced to the market in 1992 and is the latest addition to the veterinary trypanocidal list. The drug (Immiticide^®^) is also used in the treatment of heartworms in dogs, where it kills adult worms (McCall *et al.* 1994), albeit with a low margin of safety. It is registered for use against *T. b. evansi* in camels at a dose of 0·25 mg kg^−1^, but it has also been evaluated and proven efficacious against *T. b. evansi* infections in cattle (Desquesnes *et al.* 2011), goats (Gutierrez *et al.* 2008) and horses (Tamarit *et al.* 2010), although at higher dosages than that applied to camels (i.e. 0·5 mg kg^−1^ or above). Melarsomine also proved curative in cattle infected with *T. b. evansi* strains resistant to suramin (Payne *et al.* 1994). Moreover, treatment regimens with both 0·25 and 0·5 mg kg^−1^ of the drug were proven effective in curing acute and chronic *T. b. equiperdum* infections in horses, resulting in a reduction of neurological symptoms (Hagos *et al.* 2010) and offering a possible treatment for these infections. Side effects to the drug are usually mild (salivation, lacrimation, muscle tremors, increased gut motility and frequent urination), but a severe adverse reaction has also been documented (Berlin *et al.* 2010). Reports of neurological sequelae in dogs (Hettlich *et al.* 2003), albeit perhaps not analogous to the reactive encephalopathy associated with melarsoprol treatment of humans (Blum *et al.* 2001), are notable. Should the reduced neurotoxicity of melarsomine be replicated in man it might be considered as a replacement for melarsoprol, although it is doubtful that comparative clinical trials of the two arsenicals would receive ethical clearance, especially since melarsoprol is being phased out in favour of nifurtimox–eflornithine combination therapy (Simarro *et al.* 2012). The paucity of compounds that kill adult filarial worms is of note too, and, should the safety profile of melarsomine be acceptable, it could be considered for use against the human filariases.

The mode of action of melarsomine is unknown. As for other trypanocidal arsenicals, the disruption of the thiol-redox balance is a possible mechanism (Fairlamb, 2003). The drug (or, rather, its metabolite melarsen oxide) enters *T. brucei* via the same P2/*Tb*AT1 adenosine nucleoside transporter (Carter and Fairlamb, 1993; De Koning and Jarvis, 1999) and *Tb*AQP2 (Munday *et al.* 2014) that carry other melaminophenyl arsenicals and the diamidine trypanocides. Selective uptake probably accounts for most of the selective toxicity of the arsenicals (Baker *et al.* 2013). Reduction of P2/*Tb*AT1 activity is a known reason behind onset of cross-resistance between the compounds that enter via this route: trypanosomes of the *T. brucei* group resistant to melarsomine are often also less sensitive to diamidines and other arsenical drugs as melarsoprol, but not to suramin (Zhang *et al.* 1991; Pospichal *et al.* 1994). *In vitro* and *in vivo* selected melarsomine-resistant *T. b. evansi* (Suswam *et al.* 2001) revealed that the decrease in P2/*Tb*AT1 transporter activity was linked both to reduced transporter expression and changes in binding properties (Suswam *et al.* 2003). In a *T. b. brucei* strain selected for melarsomine resistance in mice the *TbAT1* gene was still present but its transcript was lost (Stewart *et al.* 2010). The lack of authentic orthologues of *Tb*AT1 and *Tb*AQP2 in *T. congolense* and *T. vivax* (see subsection *Diminazene aceturate* above) may explain why the drug is less potent against these parasites.

## DRUG RESISTANCE IN THE FIELD: DEFINITION AND EXTENT OF THE PROBLEM

Drug resistance is suspected when treatment failure occurs using standard drug dosages. However, in the field, this interpretation can be erroneous, as treatment failure can result from many factors other than the parasite's increased tolerance to drugs. For example, the presence of parasites in treated animals could correspond to a new infection rather than to recrudescence, particularly in areas of high challenge (Rowlands *et al.* 2001). Using microsatellite DNA markers to strain type *T. congolense* from cattle in Ethiopia following treatment with diminazene, essentially equal occurrences of new infection (40%) and actual relapse (37·5%) were proposed (Moti *et al.* 2015). Other causes of treatment failure not linked to true drug resistance could be related to the poor health state of the animal (e.g. malnutrition, immunosuppression, concurrent infections), or to incorrect drug use (e.g. irregular treatment or prolonged intervals between treatments), or to under-dosage. The latter can result from poor drug quality (either due to inappropriate storage or to the use of counterfeit products) (Sutcliffe *et al.* 2014), or from incorrect drug usage (wrong dilution, use of unsterilized water or erroneous dosage due to inaccurate estimation of the animal weight) (Van den Bossche *et al.* 2000; Grace *et al.* 2009). For phenanthridines, in particular isometamidium, the adverse reaction which often appears at the injection site might possibly alter drug absorption and diminish the levels of drug in circulation (Kinabo, 1993), thus determining under-dosage. It is widely believed that under-dosing could represent a major determinant in drug resistance development in the field through parasite exposure to sub-curative drug concentrations (Leach and Roberts, 1981). A similar phenomenon could derive from failure to comply with strict dose timing, which could lead to periods where sub-prophylactic drug levels are present (Leach and Roberts, 1981). Moreover, as a mutagen, homidium might also directly contribute to resistance appearance through induction of mutations in parasites that are then selected under drug pressure. Constant parasitological monitoring is necessary to distinguish treatment failure from appearance of true resistance.

In the previous section, we have outlined that there are issues associated with selected resistance to each of the drugs used against the animal trypanosomiases. Cases of resistance to veterinary trypanocides started to be reported in the field soon after their introduction, and their numbers have been increasing ever since (Delespaux *et al.* 2008*b*). A review of available literature in 2008 reported loss of efficacy of the available AAT trypanocides in at least 17 African countries (Delespaux *et al.* 2008*b*). Available data, in 2001, indicated that resistance to isometamidium was more widespread than resistance to diminazene (Geerts *et al.* 2001), however, this may no longer be so, as prevalence of resistance may change substantially over a few years (Delespaux *et al.* 2008*a*). Treatment failure against *T. congolense* and *T. vivax* infections with either of these drugs has been observed in both West (Kupper and Wolters, 1983; Pinder and Authie, 1984; Knoppe *et al.* 2006; Mungube *et al.* 2012; Vitouley *et al.* 2012) and East Africa (Mbwambo *et al.* 1988; Chitambo and Arakawa, 1992*a*; Dagnachew *et al.* 2015; Moti *et al.* 2015). More worryingly, strains of *T. congolense* resistant to both isometamidium and diminazene have been detected in several locations, including Cameroon (Mamoudou *et al.* 2008), Burkina Faso (Clausen *et al.* 1992), Ethiopia (Codjia *et al.* 1993; Afewerk *et al.* 2000; Moti *et al.* 2012), Somalia (Ainanshe *et al.* 1992) and Zambia (Sinyangwe *et al.* 2004), rendering their use as a sanative pair inoperative. These multiple resistant stocks might be the result of separate selection processes for the two drugs, as cross-resistance between diminazene and isometamidium has been considered a rare phenomenon.

Drug resistance to animal trypanocides has also been reported from outside of Africa. For example, *T. vivax* strains refractory to diminazene were identified in South America, where the compound is the first line drug to treat these infections (Desquesnes *et al.* 1995; Cadioli *et al.* 2012). Diminazene treatment failure against *T. b. evansi* infections in horses and mules in Thailand has also been reported, following decades of use (Tuntasuvan *et al.* 2003). *Trypanosoma b. evansi* strains resistant to suramin (Zhou *et al.* 2004) and quinapyramine (Zhou *et al.* 2004; Liao and Shen, 2010) have been reported in China as well as in Africa (El Rayah *et al.* 1999).

## TESTS FOR RESISTANCE DETECTION

### *In vivo* methods

Because of the confounding factors that can cause treatment failures, outlined above, methods to assess true resistance are crucial. However, reliable tests have been relatively difficult to establish for widespread use in settings where AAT is endemic. Methods such as the ‘block treatment’ approach (Delespaux *et al.* 2008*b*) have been proposed to enable identification of probable resistance in the field, whereby cattle in a particular location are split into control and treated groups and followed to first detection of parasitaemia. Broadly, the presence of resistance is measured by comparing time to parasite detection in treated *vs* the untreated controls (Eisler *et al.* 2000): the closer to the control group, the more likely the presence of resistance. However, although there are logistical advantages to such tests (e.g. no requirement for parasite isolation), they still require considerable investment in time and numbers of cattle involved (typically revisits every two weeks for 10–14 weeks, suggested group sizes of 30–80 animals), and the results are only indicative of resistance. Confirmatory trypanocide efficacy studies against veterinary trypanosomes still rely primarily on infection and treatment experiments in the natural hosts or in laboratory animals (i.e. rodents), where parasite clearance from blood following treatment is assessed by microscopy (Eisler *et al.* 2001). However, the requirement for long follow-up periods (i.e. 100 days for *in vivo* tests in ruminants and 60 days when using mice models) makes tests cumbersome, expensive and slow, as well as susceptible to the confounding factor of re-infection after successful treatment when tests are undertaken in the field. Extrapolation of rodent data to ruminants is not necessarily an accurate reflection of treatment success in large animals and, crucially, neither *T. vivax* nor many *T. congolense* strains adapt readily to propagation in mice (Eisler *et al.* 2001). Nevertheless, the single-dose mouse test is currently considered the standard test to study single or multiple resistance in *T. congolense* and *T. brucei* isolates at an accelerated rate. In spite of its being non-quantitative, the test does offer a relatively rapid (60 days) means to qualitatively assess whether parasites respond to doses of drug routinely used in veterinary practice or not. Substitution of microscopy with PCR techniques, such as the ITS1 TD PCR (Tran *et al.* 2014) for the detection of trypanosomes in blood, offers a mean to improve drug sensitivity studies using mouse models.

### *In vitro* methods

Laboratory cultivation of bloodstream form *T. brucei* transformed our ability to assess sensitivity to drugs, especially in the quantities made possible by large chemical libraries and robotic screening, resulting in new lead compounds (Nare *et al.* 2010; De Koning *et al.* 2012; Diaz *et al.* 2014). However, as previously mentioned, field isolates of livestock trypanosome species have proven difficult to establish in culture. Initial cultivation techniques required pre-cultivation over a feeder cell layer of mammalian cells, and subsequently media without the feeder layer were developed (Baltz *et al.* 1985; Hirumi and Hirumi, 1994). Recently, metabolic profiling to learn exactly what *T. brucei* use from their rich culture medium has allowed development of a refined medium for growth of these parasites (Creek *et al.* 2013). It will be of interest to use the same methods to refine the currently limited media options for *T. congolense* and *T. vivax*.

Several *in vitro* assays have been developed to determine the drug sensitivity of isolates in a faster and cheaper way than *in vivo* tests (Kaminsky and Brun, 1993). The Alamar blue test (Räz *et al.* 1997) has become the gold standard. Other assays include the drug incubation infectivity test (DIIT) (Kaminsky *et al.* 1990) and the [^3^H]hypoxanthine incorporation test (Brun and Kunz, 1989), which do not require *in vitro* adaptation of the trypanosome strain under study, and evaluate parasite viability by their ability to respectively infect mice, or incorporate tritiated hypoxanthine, following 24 h exposure to drug dilutions in a culture medium. Another option for monitoring of resistance onset for *T. congolense* has been the Drug Incubation *Glossina* Infectivity Test (DIGIT) (Clausen *et al.* 1999): drug resistant and sensitive parasites are distinguished by their ability to infect tsetse flies following their *in vitro* treatment with specific doses of trypanocidal agents.

### Molecular methods

Given the limitations in assessing drug sensitivity levels of veterinary trypanosomes, the development of molecular tests to determine parasites’ susceptibility status would be of profound importance. For *T. brucei* group parasites it has been shown that mutations in *TbAT1* and *TbAQP2* genes can underlie resistance to both melaminophenyl arsenicals and to diamidines such as pentamidine (Graf *et al.* 2015; Munday *et al.* 2015*a*, *b*). The *TbAT1* gene is also mutated in *T. brucei* group parasites (including *T. b. evansi* and *T. b. equiperdum*) when selected for diminazene resistance (Barrett *et al.* 1995; Stewart *et al.* 2010). Also, in *T. brucei* loss of the amino acid transporter *Tb*AAT6 underlies resistance to the human African trypanocide eflornithine (Vincent *et al.* 2010; Schumann *et al.* 2011; Alsford *et al.* 2012). PCR-based techniques to assess status of these resistance alleles have, therefore, been possible (Kazibwe *et al.* 2009; Graf *et al.* 2013). It has also been possible to develop non-genetic tests for resistance, such as the fluorescence-based test to assess the presence or absence of the P2/*Tb*AT1 transporter (Stewart *et al.* 2005).

For *T. congolense* and *T. vivax*, however, no reliable markers for drug resistance have yet emerged. As discussed above (see subsection *Diminazene aceturate*), the assignment of a gene named *TcoAT1* as a possible marker for diminazene resistance was erroneous. The *Mbo*II–PCR–RFLP was exploited to detect the polymorphism in an ABC-type multidrug transporter putative gene related to isometamidium resistance in *T. congolense* (Delespaux *et al.* 2005), although this link awaits validation and has not replaced the standard *in vivo* assays in West Africa (Mamoudou *et al.* 2008). However, the possibility that the intrinsic fluorescence of isometamidium could provide a useful marker for resistance to this drug, based on observations that reduced accumulation can underlie resistance, would be useful to follow up; field application would be feasible thanks to the introduction of small but robust, battery-operated fluorescence microscopes, using long-lived light-emitting diodes as fluorescent light sources (Jones *et al.* 2007).

The paucity of reliable, standardized molecular and diagnostic assays for drug resistance in animal trypanosomes relates to the diversity of infecting species and the difficulties of establishing *in vitro* cultures for most of them. Neither the basis for drug sensitivity nor drug resistance mechanisms are necessarily shared between the main species that cause AAT (*T. brucei* spp., *T. vivax, T. congolense*). Accordingly, it is essential that resistance mechanisms and mode of action models developed in one are properly investigated in the other species, rather than automatically assumed to apply.

## NEW COMPOUNDS IN THE PIPELINE

In spite of the economic importance of the veterinary trypanosomiases (in particular of AAT) and of the spreading spectre of drug resistance, new compounds for the diseases have not emerged in many years. A similar difficulty had befallen HAT in the late 20th century, which sparked the emergence of the Drugs for Neglected Diseases initiative (DNDi) and the Consortium for Parasitic Drug Development (CPDD) (Brun *et al.* 2011). These organizations were founded to fill the gap left by the pharmaceutical industry, who judged the investment required to bring new drugs forward to treat diseases of the world's poorest people had no economic rationale. In the case of HAT, the clinical development of pafuramidine, an orally available prodrug of the diamidine furamidine, was halted at an advanced stage due to unforeseen toxicity issues (Paine *et al.* 2010). Since then, DNDi have introduced a more effective combination of two older drugs, eflornithine and nifurtimox (Priotto *et al.* 2009), and brought forward the nitroimidazole fexinidazole (now in phase 2/3 trials as an oral treatment for second stage HAT) and also the benzoxaborole SCYX-7158 (AN5568), another orally available compound with the potential to treat second stage disease (Eperon *et al.* 2014). In 2011, the Global Alliance for Livestock Veterinary Medicines (GALVmed), a product development partnership supported by the UK government's Department for International Development (DFID), the Bill & Melinda Gates Foundation and the European Commission, launched a new programme aimed at the discovery of new drugs, vaccines and diagnostics for animal trypanosomiases (https://www.galvmed.org/en/livestock-and-diseases/livestock-diseases/animal-african-trypanosomosis/), on similar principles to the product development partnerships that have aimed to find new treatments for human diseases. A collaboration with Anacor Pharmaceuticals Inc. is hoping to develop and progress a separate benzoxaborole compound to that in human development (Jacobs *et al.* 2011; Steinmann *et al.* 2015) for the treatment and prevention of AAT. Several other compounds are entering the GALVmed portfolio, as they seek treatments that fulfil important features laid down in a target product profile (TPP), used to assess what properties compounds should have if they are to make an impact in AAT (Table 2).

**Table 2.** Ideal TPP of a new therapeutic and prophylactic trypanocide for animal African trypanosomiasis [from (http://www.galvmed.org)].

Attribute	Therapeutic agent	Prophylactic agent
Active ingredient	Novel agent (no cross-resistance to existing products)	Novel agent (no cross-resistance to existing products)
Indication for use	*T. congolense, T. vivax, T. brucei, T. evansi* including strains resistant to existing products	*T. congolense, T. vivax, T. brucei, T. evansi* including strains resistant to existing products
Target species	Cattle, sheep, goat & other ruminants, camels, horses, donkeys, pigs	Cattle, sheep, goat and other ruminants, camels, horses, donkeys, pigs
Route of administration	Injectable (IM, SC), oral option for sheep	Injectable (IM, SC), oral option for sheep
Formulation	Pre-formulated solution (injectable), solid bolus or suspension/solution drench (oral)	Pre-formulated solution (injectable), solid bolus or suspension/solution drench (oral)
Regimen	Single dose	Single dose
Period of protection	Not applicable	6 months
Recommended time of treatment	At first diagnosis	Before (or on) introduction to infected regions or at start of tsetse season. If also therapeutic: use at first clinical signs of disease
Expected efficacy	Absence of parasitaemia and improvement of clinical signs	Absence of parasitaemia and improvement of clinical signs
Target animal safety	No significant adverse reactions, minimal administration site reaction	No significant adverse reactions, minimal administration site reaction
Withdrawal period	Milk 0 (<7) days, meat <14 days	Milk 0 (<7) days, meat <21–28 days
Special requirements for animals	Compatible for concomitant use with common treatments	Compatible for concomitant use with common treatments
Special requirements for persons	No special precautions required	No special precautions required
Price to user	Preferably <US$ 2/dose	Preferably <US$ 2/dose
Storage requirements	Ambient temperature, ⩽40 °C/75% RH	Ambient temperature, ⩽40 °C/75% RH

IM, intramuscular; SC, subcutaneous.

Ideally, veterinary drugs, especially in resource-poor and in transhumance societies, should be of high and consistent quality, administrable in single doses sufficient to eliminate or prevent infection, be low cost with high value to the animal's owner, be active on all the relevant species of trypanosomes, have good safety profiles and, for animals destined for human consumption, ideally have very short withdrawal periods. The focus should be on classes of compounds chemically different from existing trypanocides, to minimize potential cross-resistance, and on new compounds selected for slow resistance development.

As efforts to produce new trypanocides and enhanced screening against *T. congolense* and *T. vivax* as well as *T. brucei* spp. are underway, it is becoming increasingly clear that these three species of parasites respond differently to the same compounds, a fact that is likely due to the significant differences in biochemical physiology and membrane transporters of the causative parasites. Different distribution within host tissues will also influence a drug's ability to eradicate an infection. Hence, we can assume that the development of new drugs for the animal trypanosomiases will not be straightforward, will require substantial resources to make progress over a number of years, and will be necessarily linked to increased knowledge of the genetic and phenotypic differences between the three main species of African trypanosomes.

## Concluding remarks

Since the introduction of the first veterinary trypanocides more than 60 years ago, treatment of livestock trypanosomiases worldwide has seen barely any innovation, although the available drugs have progressively become less effective and the importance of these infections has not diminished. Indeed, the growing human population and the increasing demand for food (particularly meat and milk) in the tropical and subtropical countries, where these diseases are enzootic, have elevated their importance. Moreover, control of AAT is also becoming an indispensable requisite in the context of the ‘One Health’ approach to eliminate HAT (Simo and Rayaisse, 2015). Hence, research into new curative and chemoprophylactic drugs, with a special focus on efficacy against parasite strains resistant to current treatments, is key.

The search for new veterinary trypanocides would greatly benefit from a better understanding of the biology and the metabolism of animal trypanosomes. This knowledge will be essential to elucidate the mode of action of current trypanocides, understand the molecular factors underpinning resistance, identify new drug targets and quickly screen for new leads. This, in turn, will only be possible if improved laboratory techniques to study these parasites are developed. In particular, the definition of an *in vitro* culture system for the bloodstream form of *T. vivax* must be a high priority. At the same time, current empirically formulated culture media used for other trypanosomes should be improved, to better reflect physiological availability of nutrients and make their response to (experimental) drugs more predictive of the *in vivo* situation, including the rapid detection of resistance development in the field.

While waiting for new trypanocides to become available (which may be some years away) a correct and rational use of the few already licensed drugs is paramount to ensure continuity of their effectiveness. This requires an integrated approach that includes both vector control and appropriate livestock management. Improved, more sensitive diagnostics to promptly detect infected animals, correct treatment of these with the appropriate drug, and improvement of animal general health to help their immunological response to infection, are all important actions to be undertaken that will prolong the useful lifetime of any drug. Use of trypanotolerant breeds (in Africa) and restricting the movement of potentially infected animals (in particular those harbouring mechanically transmitted trypanosomes) are other important control measures to be implemented. The use of sanative pairs (such as diminazene and isometamidium) is essential, although ineffective on its own where multiple-drug resistant trypanosomes are present.

Constant monitoring of drug use and drug resistance appearance will be crucial for correct trypanocide use and to readily improve recommendations for first use and for back up drugs to be utilized in a certain areas when resistance has been confirmed. More efforts and resources will be needed in this field, in order to better understand the extent and the distribution of the trypanosomiases and of resistance to the veterinary trypanocides. The FAO, in collaboration with the International Atomic Energy Agency (IAEA) and the framework of the Programme Against African Trypanosomosis (PAAT) are working in this direction with the launch of the ‘Atlas of tsetse and AAT’, aimed at developing a geospatial database of AAT and *Glossina* species (Cecchi *et al.* 2014). For an accurate map of drug resistance occurrence, however, improved tools for its detection in the field are needed, and their development will highly depend on a more thorough understanding of the basic processes that determine resistance onset.
